# A randomized double-blind placebo-controlled phase I/II clinical trial of a human papillomavirus therapeutic vaccine, PepCan, for reducing head and neck squamous cell carcinoma recurrence

**DOI:** 10.18632/oncotarget.28892

**Published:** 2026-06-05

**Authors:** Emily Bivens, Omar Atiq, Teresa Evans, Milan Bimali, Ginger Brown, Jasmine Crane, Nadia Darwish, Jennifer L. Faulkner, Rangaswamy Govindarajan, Aaron Johnson, Alongkorn Kurilung, Oxana Lazarenko, Yong-Chen William Lu, Keanna Marsh, Mauricio Moreno, Intawat Nookaew, Michael Robeson, Jumin Sunde, David Ussery, Emre Vural, Mindy Wilman, Mayumi Nakagawa

**Affiliations:** ^1^Department of Pathology, University of Arkansas for Medical Sciences, Little Rock, AR 72205, USA; ^2^Internal Medicine, University of Arkansas for Medical Sciences, Little Rock, AR 72205, USA; ^3^Otolaryngology – Head and Neck Surgery, University of Arkansas for Medical Sciences, Little Rock, AR 72205, USA; ^4^Biostatistics, University of Arkansas for Medical Sciences, Little Rock, AR 72205, USA; ^5^College of Medicine and Winthrop P. Rockefeller Cancer Institute, University of Arkansas for Medical Sciences, Little Rock, AR 72205, USA; ^6^Biomedical Informatics, University of Arkansas for Medical Sciences, Little Rock, AR 72205, USA

**Keywords:** human papillomavirus, head and neck cancer, therapeutic vaccine, adjuvant, clinical trial

## Abstract

Objectives: The main goals were to assess safety and efficacy (i.e., recurrence reduction).

Results: Seventeen patients were enrolled. The most common adverse events were grades 1 and 2 injection site reactions, and they occurred more frequently in the PepCan group (*p* < 0.0001). Two patients had allergic reactions (grade 2 and grade 3), at the 6th vaccination, which were considered to be a dose-limiting toxicity. No serious adverse events were reported. In the intention-to-treat analyses, 45% (5/11) had non-recurrence in the PepCan group while 80% (4/5) had non-recurrence in the placebo group (*p* = not significant). Those who received PepCan and experienced non-recurrence showed a trend of having higher new peripheral T cell immune responses to human papillomavirus type 16 E6 (*p* = 0.05). Pre-vaccination T helper type 1 cells were higher in the PepCan non-recurrence group compared to the PepCan recurrence group (*p* = 0.01).

Methods: PepCan consists of four human papillomavirus type16 E6 peptides and a *Candida* skin testing reagent. Patients with head and neck squamous cell carcinoma who had no evidence of disease after standard of care treatments were randomized at 3:1 to PepCan versus placebo (saline). Seven intradermal injections were given followed with two observational visits. Safety was assessed using CTCAE version 5, and efficacy was assessed based on not having recurrence within 2 years. In addition, immune responses and oral and gut microbiome were assessed.

Conclusions: PepCan was well tolerated. PepCan does not seem to be effective in reducing recurrence; however, the results are inconclusive given the small patient numbers.

## INTRODUCTION

The estimated new cases of head and neck squamous cell carcinoma of head and neck (HNSCC) in the United States are 60,480, and the estimated deaths are 13,150 in 2026 [1]. Worldwide, the estimated new cases are 501,968 (188,960 for larynx, 120,416 for nasopharynx, 106,316 for oropharynx, and 86,276 for hypopharynx), and estimated deaths are 269,877 (103,216 for larynx, 73,476 for nasopharynx, 52,268 for oropharynx, and 40,917 for hypopharynx) [2]. Human papillomavirus (HPV) is best known as the causative agent of cervical cancer, but it can also cause cancers at other mucosal sites, including the anus, penis, vagina, vulva, and oropharygeal squamous cell carcinoma (OPSCC), a subgroup of HNSCC. Incidences of HPV-associated OPSCC and anal cancers have increased notably [3]. The recurrence rate of HNSCC is approximately 80% over 2 years (50%–60% local recurrence, 20%–30% distant recurrence) [4, 5].

PepCan is a candidate therapeutic HPV vaccine our group developed, and it consists of human papillomavirus type 16 (HPV 16) E6 peptides and a *Candida* adjuvant (Candin^®^, Nielsen BioSciences, San Diego, CA, USA). This *Candida* is a clear extract and not heat-killed. It is known to have general immune stimulation effects and has been shown to promote interleukin-12 (IL-12) secretion *in vitro* by Langerhans cells, which is mediated by dectin-1 [6]. Because of this property, HNSCC patients regardless of HPV status were enrolled. *Candida* injection has been shown to be effective in regressing common warts [7, 8]. HPV transformation of squamous epithelium to a malignant phenotype is mediated by two early gene products, E6 and E7 [9]. Both viral proteins interact with products of cellular human tumor-suppressor genes [9]: the E6 protein can bind and promote degradation of cell-encoded p53, and the E7 protein interacts with the retinoblastoma susceptibility gene product [10–12]. Expression of E6 and E7 proteins has been shown to be necessary and sufficient for HPV-16 transformation of human cells [13–15]. While both proteins are possible candidates as components of therapeutic HPV vaccine, we chose to include E6 because T cell immune responses to E6 but not E7 has been associated with regression of cervical intraepithelial neoplasia (CIN) [16, 17]. PepCan was first tested for its ability to regress CIN [18, 19]. In this study, we examined its safety and a potential of reducing recurrence of HNSCC.

## RESULTS

### Patients and treatment

**Figure 1:**
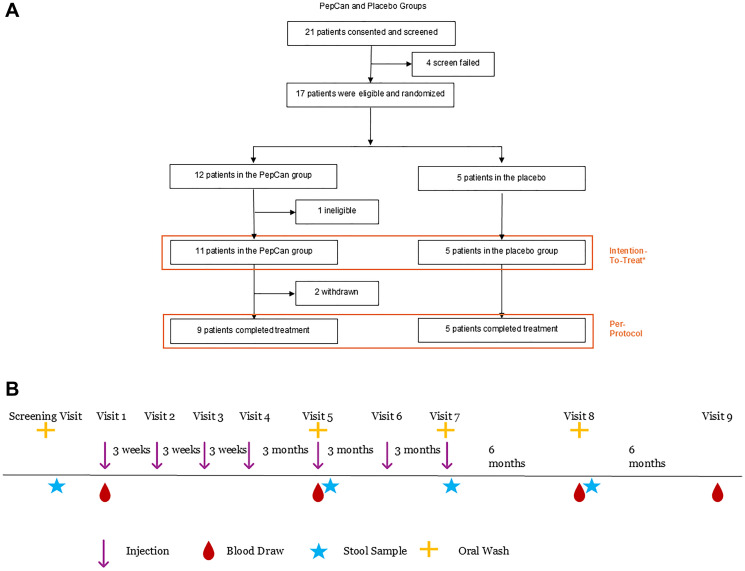
Consort diagram and the trial design. (**A**) A consort diagram summarizing patient progress during the trial. One patient in the PepCan arm was found to be “ineligible” for having had a previous recurrence. This patient was followed for safety but excluded from efficacy analysis. (**B**) The first 4 injections (PepCan or placebo) were given every three weeks, and the next 3 injections were given every 3 months. Patients had two follow-up visits six months apart after the final injection. Blood was collected at visits 1, 5, 8, and 9 for immunological assessments by ELISPOT, bulk TCR deep sequencing, and FACS analysis, and for cytokine analysis. Oral wash samples were collected at screening, visit 5, visit 7, and visit 8 and stool samples were collected at home after these visits for microbiome analyses.

Twelve patients were randomly assigned to the PepCan arm and five patients were assigned to the placebo (saline) arm (Figure 1). One patient assigned to the PepCan arm was determined to be ineligible during the study due to a previous recurrence, and this individual was included in the baseline characteristics and followed for the remainder of the study for safety. This patient was excluded from efficacy analysis. Remaining patients in the PepCan and placebo arms were included in the ITT population (*n* = 16) including two withdrawn patients who were considered to be non-responders (i.e., recurrence). These withdrawn patients were excluded from PP population (*n* = 14).

The screening questionnaire (Supplementary Figure 1) revealed that none (out of 18 patients who completed the questionnaire including one screen failure) had ever received an HPV prophylactic vaccine. The main motivation for participating was to take care of personal health for 66.7% (12/18). Most patients are either full-time workers (27.8% or 5/18), retired (27.8% or 5/18), or disabled (27.8% or 5/18). The majority’s highest education received was either high school (33.3% or 6 /18) or enrolled but did not complete college (33.3% or 6/18). Most commonly, they had 3 to 5 children (44.4% or 8/18). The number of lifetime sexual partners ranged from 1 to 50 with 44.4% (8/18) having 5 to 9 lifetime partners being most common. Twelve patients (66.7%) have reported participating in vaginal sex, four (22.2%) did not want to say, and one (5.6%) reported not having sex. Ten (55.6%) were former but not current tobacco users, and five (27.8%) were current users. Eleven (61.1%) did not drink alcohol on a regular basis while seven did (38.9%).

Their baseline characteristics were mostly well balanced, but the placebo group included only patients with squamous cell carcinoma sites of the pharynx while the PepCan group included sites of the pharynx, larynx, and oral (Table 1). The patients in the placebo group mostly had T2 disease, but the patients in the PepCan group were evenly distributed among T1 through T4 diseases. HPV status, determined using RNAscope [20], and/or P16 positivity were similar between the two treatment groups. Enrollment was halted early after 17 patients were enrolled because of vaccine peptide production failure and because of the results of another clinical trial which showed more efficacy with *Candida* alone in comparison to PepCan for treating cervical intraepithelial neoplasia 2/3 [21].

**Table 1:** Baseline characteristics of the patients

Characteristic	Overall *N* = 17	PepCan *N* = 12	Placebo *N* = 5
**Age**			
Mean (SD)	59.82 (9.82)	60.03 (8.30)	59.34 (14.00)
Median (IQR)	61.40 (13.00)	62.60 (9.55)	54.30 (23.00)
(Min., Max.)	(41.30,74.80)	(41.30,69.20)	(43.80,74.80)
**Sex**			
Male	15/17 (88.2%)	11/12 (91.7%)	4/5 (80%)
Female	2/17 (11.8%)	1/12 (8.3%)	1/5 (20%)
**Race**			
White	12/17 (71%)	7/12 (58%)	5/5 (100%)
Black	4/17 (24%)	4/12 (33%)	0/5 (0%)
Other	1/17 (5.9%)	1/12 (8.3%)	0/5 (0%)
**Ethnicity**			
Non-Hispanic	16/17 (94%)	11/12 (92%)	5/5 (100%)
Hispanic/Latino	1/17 (5.9%)	1/12 (8.3%)	0/5 (0%)
**WBC**			
Mean (SD)	5.03 (1.55)	5.20 (1.52)	4.64 (1.70)
Median (IQR)	5.04 (2.05)	5.32 (1.69)	4.33 (1.98)
(Min., Max.)	(2.96,8.24)	(2.96,8.24)	(3.00,7.22)
**Hemoglobin**			
Mean (SD)	11.63 (1.81)	11.94 (1.91)	10.88 (1.43)
Median (IQR)	12.10 (2.50)	12.30 (1.73)	11.10 (2.70)
(Min., Max.)	(8.30,15.40)	(8.30,15.40)	(9.30,12.30)
**Hematocrit**			
Mean (SD)	35.55 (5.56)	36.63 (5.73)	32.96 (4.64)
Median (IQR)	36.50 (8.60)	37.50 (6.15)	35.00 (8.90)
(Min., Max.)	(26.40,46.90)	(26.40,46.90)	(27.90,37.00)
**BMI**			
Mean (SD)	22.17 (3.87)	22.30 (3.82)	21.86 (4.41)
Median (IQR)	22.65 (4.91)	22.99 (4.51)	20.67 (4.91)
(Min., Max.)	(15.82,29.25)	(15.82,29.25)	(16.46,27.76)
HPV^*^			
Negative	1/1 (100%)	0/0 (−%)	1/1 (100%)
Unknown	16	12	4
**P16**			
Positive	7/12 (58%)	4/9 (44%)	3/3 (100%)
Negative	5/12 (42%)	5/9 (56%)	0/3 (0%)
Unknown	5	3	2
**HPV/P16**			
Positive	7/13 (54%)	4/9 (44%)	3/4 (75%)
Negative	6/13 (46%)	5/9 (56%)	1/4 (25%)
Unknown	4	3	1
**Prior treatment**			
Surgery	17/17 (100%)	12/12 (100%)	5/5 (100%)
Chemotherapy	16/17 (94%)	11/12 (92%)	5/5 (100%)
Radiation	17/17 (100%)	12/12 (100%)	5/5 (100%)
**Site**			
Pharynx	11/17 (65%)	6/12 (50%)	5/5 (100%)
Larynx	3/17 (18%)	3/12 (25%)	0/5 (0%)
Oral	3/17 (18%)	3/12 (25%)	0/5 (0%)
**Tumor**			
T1	3/17 (18%)	3/12 (25%)	0/5 (0%)
T2	6/17 (35%)	2/12 (17%)	4/5 (80%)
T3	4/17 (24%)	3/12 (25%)	1/5 (20%)
T4	4/17 (24%)	4/12 (33%)	0/5 (0%)
**Lymph node**			
N0	2/17 (12%)	1/12 (8.3%)	1/5 (20%)
N2	10/17 (59%)	8/12 (67%)	2/5 (40%)
N1	3/17 (18%)	1/12 (8.3%)	2/5 (40%)
N3	2/17 (12%)	2/12 (17%)	0/5 (0%)
**Metastasis**			
M0	17/17 (100%)	12/12 (100%)	5/5 (100%)

^*^HPV testing was performed using in situ hybridization stains using the RNAscope method for high risk and low risk HPVs [20].

### Safety

Injection-related commonly occurring adverse events (AEs) are summarized in Table 2, and all AEs, regardless of relatedness to treatments are summarized in Supplementary Table 1. Dose-limiting toxicities (DLTs) occurred in two patients. One patient experienced grade 2 allergic reaction, after receiving the 6th injection, characterized by itching. He responded well to diphenhydramine. The other patient experienced a grade 3 allergic reaction after the 6th injection. He responded well to diphenhydramine and steroids. These patients did not receive the 7th and final vaccination. The most common AE was grades 1 and 2 injection site reactions which occurred only with the PepCan group (23/72 injections) but not with the placebo group (*p* < 0.0001). The descriptions of 3 grade 1 “vaccine complication” in the PepCan group included “a small knot about a size of a pencil eraser after the 3rd vaccination injection site on the right forearm”, “injection site with mild redness and swelling”, and “2.2 cm of redness around injection site for 4th vaccination”. One grade 2 “vaccine complication” in the PepCan group was described as “patient reported that the redness and itching stopped after a couple of days, but the knot remained.” One grade 1 “vaccine complication” in the placebo group was described as “patient reports swelling and redness for 1 week after vaccination #4, and also described it as ‘bug bite-like’. Symptoms were not self-treated.” Most other AEs such as headache and vaccination complication occurred more commonly in the PepCan group, but not significantly.

**Table 2:** Injection-related adverse events

CTCAE v5 system organ class	CTCAE v5 AE term	CTCAE v5 AE severity scale grade											
		PepCan *n* = 72						Placebo *n* = 35					
		1	2	3	4	5	Total	1	2	3	4	5	Total
General Disorders and Site Administration Conditions	Injection site reaction	20	3	–	–	–	23	–	–	–	–	–	–
	Fatigue	–	–	–	–	–	–	1	–	–	–	–	1
Immune System Disorders	Allergic reaction	–	1	1	–	–	2	–	–	–	–	–	–
Injury, Poisoning and Procedural Complications	Vaccination complication	3	1	–	–	–	4	1	–	–	–	–	1
Metabolism and Nutrition Disorders	Hypokalemia	1	–	–	–	–	1	–	–	–	–	–	–
Nervous System Disorders	Dizziness	1	–	–	–	–	1	–	–	–	–	–	–
	Headache	2	–	–	–	–	2	–	–	–	–	–	–
Skin and Subcutaneous Tissue Disorders	Pruritus	–	1	–	–	–	1	–	–	–	–	–	–

≥ grade 2 allergic reactions were considered to be dose-limiting toxicities. Abbreviation: N: number of injections.

### Efficacy

Patients with non-recurrence over the ten visits (the screening and the 9 study visits over two years) were considered to be responders while patients with recurrence were considered as non-responders (Table 3). Analysis of the ITT group revealed non-recurrence in 45% (5 /11) of patients in the PepCan group (95% confidence interval (CI), 0.17 to 0.77) and 80% (4/5) non-recurrence for the placebo group (95% CI, 0.28 to 0.99). Analysis of the PP group revealed non-recurrence in 56% (5/9) of the PepCan group (95% CI, 0.21 to 0.86) and non-recurrence in 80% (4/5) of the placebo group (95% CI, 0.28 to 0.99) Neither comparison was significant with *p*-values of 0.31 and 0.58, respectively. Unexpectedly, the placebo group showed higher non-recurrence rate; but the results are inconclusive given the small number of patients enrolled.

**Table 3:** Outcomes

Characteristic	Overall *N* = 17	PepCan *N* = 12	Placebo *N* = 5
**Outcome**			
Non-recurrence	9/17 (53%)	5/12 (42%)	4/5 (80%)
Recurrence	5/17 (29%)	4/12 (33%)	1/5 (20%)
Withdrawn	2/17 (12%)	2/12 (17%)	0/5 (0%)
Ineligible	1/17 (5.9%)	1/12 (8.3%)	0/5 (0%)
**Outcome (ITT)^1^**			
Non-Recurrence	9/16 (56%)	5/11 (45%)	4/5 (80%)
Recurrence	7/16 (44%)	6/11 (55%)	1/5 (20%)
**Outcome (PP)^2^**			
Non-Recurrence	9/14 (64%)	5/9 (56%)	4/5 (80%)
Recurrence	5/14 (36%)	4/9 (44%)	1/5 (20%)

Efficacy was assessed as “non-recurrence” within 2 years from beginning the study. ^1^The ITT analysis excluded an “ineligible” subject. ^2^The PP analysis included patients who completed the study, and excluded those who were “ineligible” or “withdrawn”.

### Immune responses

#### Enzyme-linked immunospot (ELISPOT) assay

Representative results are shown in Supplementary Figure 2. ELISPOT analysis revealed no difference between the percentage of patients who had anti-HPV 16 E6 response prior to vaccine in the PepCan and placebo arms. Likewise, no difference was found in response prior to vaccine for PepCan non-recurrence and recurrence groups. No difference was found in the development of a new response to HPV 16 E6 in at least one region between PepCan and placebo groups. However, there was a trend of more patients in the PepCan non-recurrence group (5 of 5 for ITT and PP) developing a new response for HPV 16 E6 after vaccination compared to patients in the PepCan recurrence group (1/5 for ITT (*p* = 0.05) and 0/3 for PP (*p* = 0.02)).

#### Fluorescence-activated cell sorting (FACS) analysis

**Figure 2:**
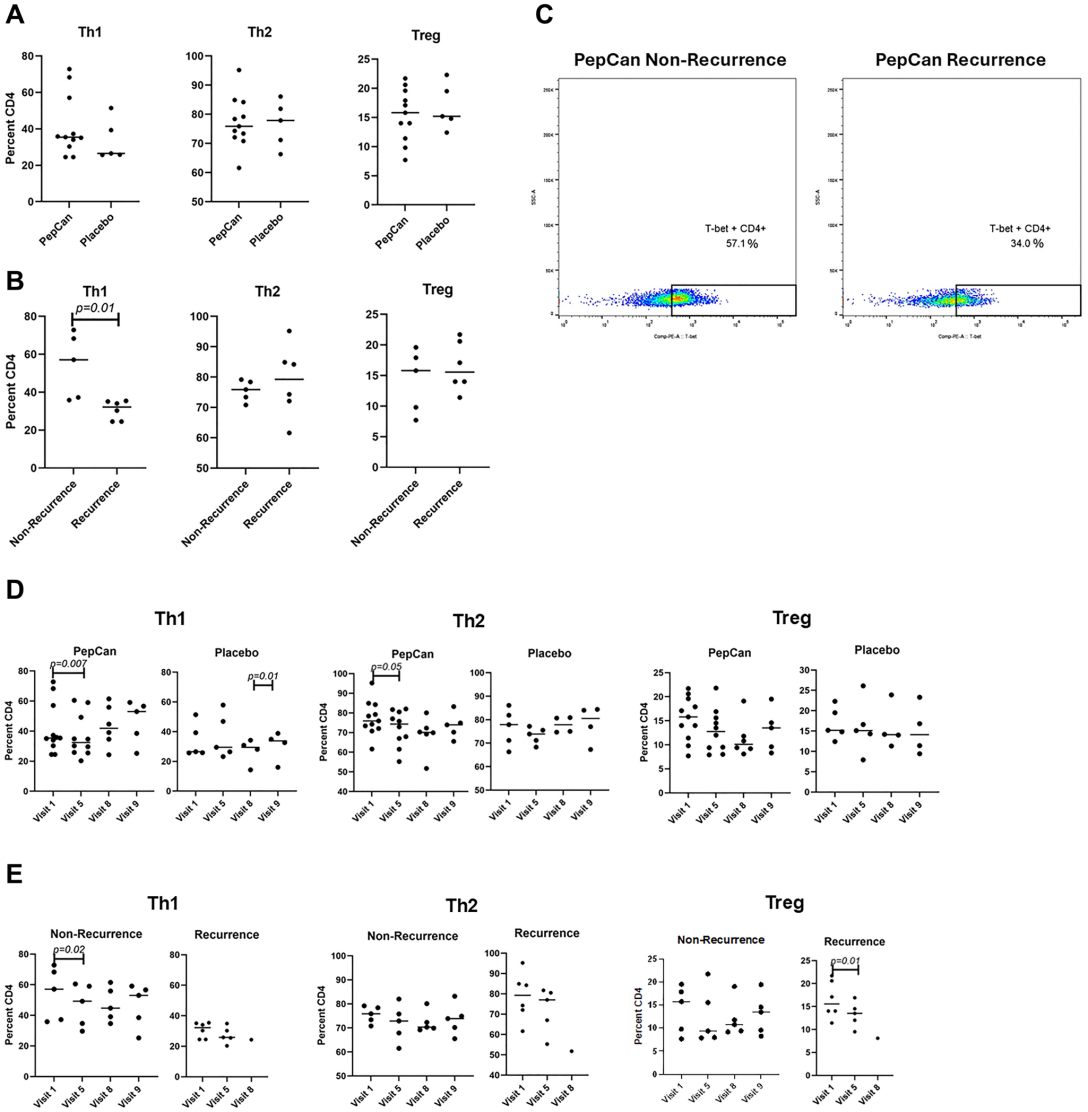
FACS analyses of peripheral immune cells ITT. (**A**) Analyses of Th1, Th2, and Treg at visit 1 (pre-vaccination) between the PepCan and placebo groups. (**B**) Analyses of Th1, and Th2, and Treg also at visits 1 between the PepCan non-recurrence and the PepCan recurrence groups. (**C**) Representative raw data plots for Th1 results for a PepCan non-recurrence (Patient 17) and a PepCan recurrence (Patient 11) patients. (**D**) Analyses of Th1, and Th2, and Treg also at visits 1, 5, 8, and 9 between the PepCan and placebo groups. (**E**) Analyses of Th1, Th2, and Treg between non-recurrence PepCan group and recurrence PepCan group at visits 1, 5, 8, and 9. The sample size is 16.

Comparisons were made of pre-vaccination levels between PepCan and placebo groups and between PepCan non-recurrence and PepCan recurrence groups. (Figure 2A for ITT and Supplementary Figure 3A for PP). The ITT analysis revealed significantly higher T-helper type 1 (Th1) cells in the PepCan non-recurrence group compared to PepCan recurrence group pre-vaccination (Figure 2B, 2C, *p* = 0.01). The results were similar for the PP analysis (Supplementary Figure 3B, *p* = 0.04). When these groups were compared across visits 1 (pre-vaccination), 5, 7, and 8, some statistically significant changes were noted. However, they may not be biologically significant based on the data point distribution (Figure 2 D, 2E for ITT and Supplementary Figure 3C, 3D for PP).

#### T Cell Receptor (TCR) repertoire analysis

Comparisons between PepCan and placebo groups and between PepCan non-recurrence and PepCan recurrence groups were made in ITT (Supplementary Figure 4A–4D) and PP (Supplementary Figure 4E–4H) populations. Data are presented as the total number of templates (i.e., the total number of putatively vaccine-specific T cells; Supplementary Figure 4A, 4B, 4E, 4F) and as a fraction of total cells (Supplementary Figure 4C, 4D, 4G, 4H). No differences were found.

### Cytokine analysis

In the ITT analysis, the mean interleukin-5 (IL-5) level was significantly lower in the PepCan group compared to placebo group (Supplementary Figure 5A) (Difference, −227.91; 95% CI, −372.88 to −82.95; *p* < 0.01). For monocyte chemotactic protein 1 (MCP-1, also known as monocyte chemotactic activating factor (MCAF)) and stromal-derived cell factor-1α (SDF-1α), the interaction effect was retained suggesting that the treatment effect was moderated by study visit. The mean MCP1 level was significantly lower in the PepCan group compared to the placebo group for visit 1 (Difference, −34.38; 95% CI: −56.76 to −12.00; *p* < 0.01). Other significant changes in SDF-1α and B-cell activating “factor” (BAFF/TNFSF13B) among placebo-treated patients were found for some visit comparisons (Supplementary Figure 5A).

As to the comparisons between the PepCan non-recurrence and recurrence group ITT, the mean IL-5 level was significantly higher among the PepCan non-recurrent patients (Supplementary Figure 5B) (Difference, 215.49; 95% CI, −86.81 to 344.17; *p* < 0.01). The interaction effect was retained for Interleukin-1 receptor agonist (IL-1RA), IL-12 (p40), and BAFF/TNFSF13B for some visit comparisons (Supplementary Figure 5B).

For PP analysis comparing PepCan and the placebo groups, the only significance retained was that for MCP1. When comparing PepCan non-recurrence and recurrence groups (Supplementary Figure 5C), the mean chitinase-3-like 1 level was significantly lower at visit 5 compared to visit 1 (Difference, −3457.98; 95% CI, −5401.79 to −1514.16; *p* < 0.01), and the interaction effect was retained for interleukin-28A/interferon-λ2 (IL-28A/IFN-λ2) and MMP-1 (Supplementary Figure 5D).

### Microbiome

#### Stool

The compositions of stool bacteria at phylum and species are level are shown in Supplementary Figure 6A, 6B respectively for metagenomics. More diverse fecal microbiome and/or certain bacterial taxa have been associated with better outcomes for cancer patients treated with checkpoint inhibitors [22–27], and certain gut bacterial taxa have been associated with clinical responses to chimeric antigen receptor T cell therapies [28, 29]. Therefore, we compared alpha diversity between PepCan and placebo groups (Supplementary Figure 6C) and between PepCan non-recurrence and PepCan recurrence groups (Supplementary Figure 6D) prior to vaccination. We also examined differentially abundant taxa between these groups. Furthermore, alpha diversity was compared and differentially abundant taxa were examined before (at visit 1) and after (at visit 8) vaccination. No significant results were found. Analogous results of amplicon-based rRNA sequencing are shown in Supplementary Figure 7A–7D.

#### Oral wash

The compositions of oral wash bacteria at phylum and species are level are shown in Supplementary Figure 7E, 7F for amplicon-based rRNA sequencing. The comparisons of alphas diversity between PepCan and placebo groups (Supplementary Figure 7G) and between PepCan non-recurrence and PepCan recurrence (Supplementary Figure 7H) are also shown, but there were no significant comparisons.

## DISCUSSION

The main goals of this study were to assess safety and efficacy, i.e., PepCan’s ability to reduce HNSCC recurrence, and HNSCC patients with no evidence of disease after having completed standard treatments were enrolled. At present, there are two other clinical trials of HPV therapeutic vaccine trials treating HNSCC patients that require participants to have no evidence of disease. These studies utilized mRNA (NCT03418480) or dendritic cell targeting (NCT06007092). Of these, the mRNA study includes an arm for recurrent disease. Likewise, there are several other types of HPV therapeutic vaccines in clinical trials, but their focus is on the treatment of existing HPV or HNSCC malignancy. There are DNA-based (NCT03162224) [30], mRNA-based (NCT04534205), cell based (NCT04084951) [31], viral vector-based (NCT04180215), peptide-based (NCT02426892) [32], and bacterial vector-based (NCT02002182).

Previous studies which used PepCan to treat CINs demonstrated safety [18, 19, 21]. The current study also showed that PepCan is safe as there were no serious adverse events reported throughout the study. However, there were two DLT events in this study which were grade 2 and 3 allergic reactions, but no DLTs were reported in the previous studies [18, 19, 21]. Previous studies administered four PepCan injections while the current study administered seven. A larger number of injections were given to HNSCC patients since they had established cancer while patients in previous studies only had precancer. Both DLT events occurred at the 6th injection. Therefore, larger number of injections may predispose to having allergic reactions. The most common injection-related AE was injection site reaction which was significantly more frequent in the PepCan group compared to the placebo group (*p* < 0.0001).

Unexpectedly, non-recurrence rate was higher in the placebo group (4/5 or 80% for ITT and PP) compared to the PepCan group (5/11 or 45% for ITT and 5 of 9 or 56% for PP). Some differences in the baseline characteristics such as most patients in the placebo group having T2 disease may have contributed to the unexpected outcome; however, this study is inconclusive because only a small number of patients were enrolled. The expected outcome of the study used for the power calculation (see Methods) was to reduce the recurrence rate from 80% [4, 5] to 44% in the PepCan group. This is very close to the ITT non-recurrence rate of 45%.

Comparison of pre-vaccination T cell responses to HPV 16 E6 showed no difference between the PepCan and placebo groups. Given that most patients did not have HPV-positive OPSCC, the pre-vaccination HPV 16 E6 responses may be responses to infection to anogenital sites. There was a trend of new post-vaccine responses to HPV 16 E6 being higher in the PepCan non-recurrence group compared to the PepCan recurrence group (*p* = 0.05 for ITT and *p* = 0.02 for PP) suggesting that the ability to mount T cell responses may be related to clinical efficacy.

There were no differences in the pre-vaccination levels of Th1, T-helper type 2 (Th2), and regulatory T (Treg) cells between the PepCan and placebo group (Figure 2A and Supplementary Figure 3A). However, Th1 cells were significantly higher in the PepCan non-recurrence group compared to the recurrence group (Figure 2B and Supplementary Figure 3B, *p* = 0.01 for ITT and *p* = 0.04 for PP). Therefore, Th1 polarization appears to be associated with vaccine response. This make sense in that *Candida* is known to promote IL-12 production [6], and an earlier study using PepCan to treat CIN demonstrated increase in Th1 cells after vaccination [19].

Cytokine analysis showed significant difference for IL-5 and MCP1. The IL-5 levels were found to be significantly higher in the placebo group compared to the PepCan and for the PepCan non-recurrence group compared to the PepCan recurrence group suggesting better clinical outcomes with elevated IL-5 levels (Supplementary Figure 5). The biological significance remains uncertain as the significant increase in IL-5 was found only for the ITT and not PP population. A lower level in the PepCan group would have made more sense as IL-5 is associated with Th2 cells while PepCan is associated with Th1 cells [33, 34]. MCP1 was higher in placebo compared to PepCan at visit 1 for both the ITT and the PP populations. While not statistically significant, the placebo group showed better outcomes than the PepCan group. This may be explained by the role MCP1 plays in the chemotaxis of memory lymphocytes [35].

We examined differentially abundant taxa and alpha diversity of the stool samples since it is well known that it influences outcomes of immunotherapy [22–27]. No significant results were found. Analogous analyses using the oral wash samples did not yield any significant results either likely due to small number of patients enrolled.

The limitations of this study were (1) the small number of patients enrolled, (2) fewer blood, stool, and oral wash samples collected from the recurrence group compared to the non-recurrence group, and (3) inclusion of HPV-positive and negative patients. Fewer number of samples were due to patients with recurrence completing and exiting the study at the time of recurrence. Therefore, they did not necessarily finish all planned study visits.

As the Phase II study of comparing the efficacy of PepCan and *Candida* in regressing CIN unexpectedly showed better outcome with *Candida* (NCT02481414) [21], we have initiated a new clinical trial enrolling HNSCC patients with no evidence of disease after standard therapies using *Candida* to reduce recurrence (NCT05952934).

In conclusion, this study demonstrated that seven injections of PepCan are well tolerated although DLT did occur when more injections were given compared to earlier studies [18, 19, 21]. Efficacy could not be assessed given the small number of patients enrolled. There was a trend of new T cell response to HPV 16 E6 protein being higher in the non-recurrence group in comparison to recurrence group, and circulating prevaccination Th1 cells were higher in the non-recurrence group (Figure 2B and Supplementary Figure 3B).

## MATERIALS AND METHODS

### Patients

Recruitment for this study took place between October 28, 2019 and June 29, 2023. Inclusion criteria were being men or women aged 18 years or older, having completed curative therapy (surgery and/or radiation and/or chemotherapy) within the previous 120 days for HNSCC of any primary site, and having achieved no evidence of disease based on clinical and/or radiographic evaluations. Since *Candida* was known to have a general immune stimulating effects, patients were enrolled regardless of their HPV status [6]. After signing a written informed consent, patients (*n* = 21) were screened, and those who met the inclusion criteria and returned a stool sample (*n* = 17) were eligible for vaccination. They were randomly assigned to the PepCan or placebo (saline) arm at a 3:1 ratio as shown in a Consort diagram (Figure 1). Exclusion criteria included a positive urine pregnancy test, being pregnant or attempting to become pregnant, breastfeeding, allergy to *Candida*, a history of severe asthma, having previously received PepCan, or a history of recurrent HNSCC. Informed consent was obtained from each patient. The study adhered to the Declaration of Helsinki, was approved by the Institutional Review Board (217672), and was prospectively registered at ClinicalTrials.gov (NCT03821272).

### Vaccine composition and delivery

PepCan consists of four current good manufacturing production-grade synthetic peptides covering the HPV 16 E6 protein (amino acid (aa)1-45, 46-80, 81-115, and 116-158) (CPC Scientific, San Jose, CA, USA; Curia, Albany, NY, USA). Lyophilized peptides (50 μg per peptide) were reconstituted with sterile water and mixed in a syringe with 0.3 mL of *Candida* (Candin^®^, Nielsen Biosciences, San Diego, CA, USA). Candin is an extract of *Candida*, and is not heat-killed nor fixed. The PepCan mixture was administered intradermally in any limb, but most often in the anterior forearm. For the placebo arm, an equal volume of normal saline was administered.

### Trial design and end points

This clinical trial (University of Arkansas for Medical Sciences Institutional Review Board approval #217672) was a randomized, double-blind, placebo-controlled, single-center, Phase I/II study in which patients were assigned to PepCan or placebo at a 3:1 ratio using a computer-generated randomization scheme created by the study statistician. The patients and all study personnel were blinded to which vaccine the patients received, except for the research pharmacists who assigned patients to interventions. Only the study “statistician” and the study pharmacists had access to the randomization scheme. At the screening visit, the patients were given a questionnaire which asked whether they received HPV prophylactic vaccination, their motivations for participation, employment, education, number of children, number of lifetime sexual partners, type of sexual activities, and use of alcohol, tobacco or other nicotine products. After enrollment, the first four injections were given 3 weeks apart while the last three injections were given 3 months apart. Then, the patients returned for observational visits 6 and 12 months after the last injection. The primary endpoint was safety, which was assessed from the time of informed consent until visit 9 or until recurrence according to the National Cancer Institute Common Terminology Criteria for Adverse Events (CTCAE) Version 5. The DLTs were defined as grade 2 or above allergic/autoimmune reactions and any grade 3 or above AEs. The secondary endpoint was clinical response, which was assessed by lack of recurrence within two years.

### Immunological assessments

#### ELISPOT assay

Peripheral immune responses were examined by interferon-ɣ ELISPOT assay, bulk TCR deep sequencing, and immune profiling. T cell lines were established from blood draws from visits 1, 5, 8, and 9 as described previously [21]. In short, peripheral blood mononuclear cells (PBMCs) were isolated from heparinized whole blood using a Ficoll density-gradient centrifugation method, separated into CD14+ monocytes and CD14-depleted PBMCs, and cryopreserved. Autologous dendritic cells were established by growing monocytes in the presence of granulocyte monocyte-colony stimulating factor (50 ng/mL, Sanofi-Aventis, Paris, France) and recombinant interleukin-4 (100 U/mL, R&D Systems, Minneapolis, MN) for seven days, and were matured by 48-hour culture in wells containing irradiated mouse L-cells expressing CD40 ligands. CD3 T cells were magnetically selected (Pan T Cell Isolation Kit II, Miltenyi Biotec, Auburn, CA) from CD14-depleted PBMCs. HPV 16 E6- and E7-specific CD3 T cell lines were established by *in vitro* stimulation of CD3 cells for seven days with autologous dendritic cells pulsed with E6-glutathione *S*-transferase and E6 expressing recombinant vaccinia virus or E7-glutathione S-transferase and E7 expressing recombinant vaccinia virus [36, 37]. *In vitro* stimulation was repeated for an additional seven days.

ELISPOT assays were performed in triplicate using overlapping peptides covering the ten regions (aa1-25, 16-40, 31-55, 46-70, 61-85, 76-100, 91-115, 106-130, 121-145, and 136-158) within the E6 protein of HPV 16, as previously described [21]. Briefly, 96-well plates (MultiScreen-MSIPS 4510 plates, Sigma-Aldrich, St. Louis MO) were treated with 35% ethanol in sterile phosphate-buffered saline and rinsed immediately. They were subsequently coated with 5 μg/ml of primary anti-interferon-ɣ monoclonal antibody (1-D1K, Mabtech, Sweden), washed, and blocked. Then, 2.5 × 10^4^ CD3 T cells were added per well in triplicate along with pooled peptides (10 μM per peptide). Each peptide pool contained 3 overlapping HPV 16 E6 15-mer peptides. For example, the E6 1-25 pool contained E6 1-15, E6 6-20, and E6 11-25 peptides. Negative control wells contained media only, and positive control wells contained phytohaemagglutinin at 10 μg/mL, (Remel, Lenexa, KS). Plates were incubated for 24 hours at 37^o^C with 5% carbon dioxide. After washing, a secondary biotin-conjugated anti-Interferon-ɣ monoclonal antibody (7-B6-1, Mabtech, Nacka Strand, Sweden) was added at 1 μg/ml and incubated for 2 hours. After washing, streptavidin-horseradish peroxidase (Mabtech) was added at a dilution of one to 750 and incubated at room temperature for one hour. Wells were washed and coated with 3,3′,5,5′ tetramethylbenzidine (Mabtech) for 10 to 30 minutes and allowed to develop at room temperature until spots emerged. The plates were then rinsed in water and allowed to dry overnight. Peptide pools with spot forming units twice or greater to the media only control were considered to be positive. The ratio between spot forming units in peptide wells to the spot forming units in the media control is called positivity index.

#### FACS analysis

Immune profiling measuring Th1 cells, Th2 cells, and Tregs, was also performed using FACS with blood samples drawn at visits 1, 5, 8, and 9 using previously established methods [18, 19] with minor modifications. To analyze Th1 cells (CD4- and Tbet-positive), Th2 cells (CD4- and GATA3-positive), and Tregs (CD4-, CD25-, and FoxP3-positive), thawed PBMCs were treated with Fc Receptor Blocker (Innovex, Maple Plain, MN) and stained using fluorescein isothiocyanate-labeled anti-human CD4 (Thermo Fisher Scientific, Hillsboro, OR), phycoerythrin-labeled anti-human/mouse T-bet (Thermo Fisher Scientific), allophycocyanin -eFluor-labeled anti-human CD25 (clone BC96, Thermo Fisher Scientific), allophycocyanin-labeled anti-human Foxp3 (Thermo Fisher Scientific), and phycoerythrin -Cy7 labeled anti-human/mouse GATA3 (BD, Franklin, NJ). Cells were initially stained with antibodies for surface markers CD3, CD4, and CD25. Intracellular staining for T-bet, GATA3, and Foxp3 was performed using the Foxp3 staining kit (Thermo Fisher Scientific). Live/dead staining 7-AAD (BD) was used to exclude dead cells. Stained cells were analyzed using FACSCelesta (BD) available in the University of Arkansas for Medical Sciences Microbiology and Immunology Flow Cytometry Core Laboratory, and FlowJo (BD) was used to analyze the data.

#### TCR repertoire analysis

Bulk deep sequencing of polymerase chain reaction (PCR)-amplified TCR β CDR3 regions using genomic DNA from PBMCs was performed (Adaptive Biotechnologies, Seattle, WA) [38]. Using bias-controlled V and J gene primers, the rearranged V(D)J segments were amplified and sequenced. A clustering algorithm was used to correct for sequencing errors, and the V(D)J segments were annotated according to the International ImMunoGeneTics Collaboration [39, 40] to identify the V, D, and J genes that contributed to each rearrangement. A mixture of synthetic TCR analogs was used in PCR to estimate the number of cells bearing each unique TCR sequence [41]. Putatively vaccine-specific T cells were identified by comparing post-vaccination PBMC sample to the pre-vaccination PBMC sample using a beta-binomial model [42] under the immunoSEQ analyzer (Adaptive Biotechnologies). The results were obtained for the numbers of clonotypes as well as the fractions of total T cells.

### Cytokine assessments

To identify potential biomarkers predictive of vaccine response and to examine the effects of vaccination, plasma samples for 81 plasma cytokines were analyzed using methods described previously [18]. As our pilot data demonstrated that approximately one third of cytokine levels were significantly different when the blood samples were processed at 2 hours versus 1 hour [18], we used plasma samples that were processed within 1 hour of blood draw. The quantities of β-nerve growth factor (β-NGF), cutaneous T cell-attracting chemokine (CTACK), eotaxin, basic fibroblast growth factor (FGF basic), granulocyte-colony stimulating factor (G-CSF), granulocyte monocyte-colony stimulating factor (GM-CSF), C-X-C motif chemokine ligand 1 (CXCL1, also known as GRO-α), hepatocyte growth factor (HGF), interferon-α2 (IFN-α2), interferon-γ (IFN-αγ), interleukin-1α (IL-1α), interleukin-1β (IL-1β), IL-1RA, interleukin-2 (IL-2), interleukin-2Rα (IL-2Rα), interleukin-3 (IL-3), interleukin-4 (IL-4), IL-5, interleukin-6 (IL-6), interleukin-7 (IL-7), interleukin-8 (IL-8), interleukin-9 (IL-9), interleukin-10 (IL-10), IL-12 (p70), IL-12 (p40), interleukin-13 (IL-13), interleukin-15 (IL-15), interleukin-16 (IL-16), interleukin-17A (IL-17A), interleukin-18 (IL-18), IFN-γ-induced protein 10 (IP-10), leukemia inhibitory factor (LIF), monocyte-colony stimulating factor (M-CSF), MCP-1 (also known as MCAF), monocyte chemotactic protein 3 (MCP-3), macrophage migration inhibitory factor (MIF), C-X-C motif chemokine ligand 9 (CXCL9, also known as MIG), macrophage inflammatory protein1α (MIP-1α), macrophage inflammatory protein-1β (MIP-1β), platelet-derived growth factor subunit B (PDGF-BB), regulated on activation, normal T-cell expressed and secreted (RANTES), stem cell factor (SCF), stem cell growth factor β (SCGF-β), SDF-1α, tumor necrosis factor α (TNF-α), tumor necrosis factor β (TNF-β), tumor necrosis factor-related apoptosis inducing ligand (TRAIL), and vascular endothelial growth factor (VEGF) were determined using a commercially available Bio-Plex 48-plex kit (Bio-Rad Laboratories) according to the manufacturer’s instructions using a Bio-Plex 200 instrument (Bio-Rad Laboratories). The quantities of a proliferation-inducing ligand (APRIL/TNSFS13), BAFF/TNFSF13B, soluble CD30 (sCD30/TNFRSF8), soluble CD163 (sCD163), chitinase-3-like 1, glycoprotein 130/soluble interleukin-6 receptorβ (gp130/sIL-6Rβ), interferon-β (IFN-β), soluble interleukin-6 receptorα (sIL-6Rα), interleukin-11 (IL-11), interleukin-19 (IL-19), interleukin-20 (IL-20), interleukin-22 (IL-22), interleukin-26 (IL-26), interleukin-27 (IL-27, p28), IL-28A/IFN-λ2, interleukin-29/interferon-λ1 (IL-29/IFN-λ1), interleukin-32 (IL-32), interleukin-34 (IL-34), interleukin-35 (IL-35), tumor necrosis factor superfamily member 14 (LIGHT), MMP-1, matrix metalloproteinase-2 (MMP-2), matrix metalloproteinase-3 (MMP-3), osteocalcin, osteopontin (OPN), pentraxin-3, soluble tumor necrosis factor-receptor 1 (sTNF-R1), sTNF-R2, thymic stromal lymphopoietin (TSLP), and tumor necrosis factor-like weak inducer of apoptosis (TWEAK/TNSFS12) were determined separately using another Bio-Plex inflammation kit. Transforming growth factor-β1 (TGF-β1), transforming growth factor-β2 (TGF-β2), and transforming growth factor-β3 (TGF-β3) were determined separately using Bio-Plex TGF-β kit which included an acid treatment.

### Microbiome assessments

Oral wash samples were collected in clinic at screening visit and visits 5, 7, and 8, and fecal samples were collected by patients at home using the OMNIgene.GUT tubes (DNA Genotek, USA) following the same visits. The oral wash samples were collected using 10 mL of mouth wash which was swished in mouth for 30 seconds. DNA was extracted using ZymoBIOMICS DNA Miniprep Kit (Zymo Research, Irvine, CA, USA), was aliquoted, and quality was checked by TapeStation (Agilent, Santa Clara, CA) in the University of Arkansas for Medical Sciences DNA Sequencing Core Facility. Stool DNA samples were analyzed using a flow cell (R10.4.1) on a MinION Mk1B sequencer (Oxford Nanopore Technologies, New York, NY, USA), and amplicon-based rRNA sequencing (Argonne National Laboratory, Lemont, IL). The metagenomics data were processed using our coverage-based analysis for identification of microbiome (CAIM) software [43]. Oral wash DNA was analyzed using the amplicon-based method only due to high human DNA content.

A positive control utilized in this study was mock vaginal microbial communities composed of a mixture of genomic DNA from the American Type Culture Collection (Manassas, VA, USA) and Zymo Research. The negative control was OMNIgene.GUT preservation solution without the sample as blank extraction [44]. Controls and the extracted DNA were amplified and analyzed using the 16S rRNA gene on an Illumina MiSeq sequencing platform [45]. The same volume of DNA was used for each reaction, and then normalized at the PCR pooling step. This ensures that equal amounts of each amplified sample are added to the sequencing pool. Paired-end reads from libraries with ~250-bp inserts were generated for the V4 region using the barcoded primer set: 515FB: 5′-GTGYCAGCMGCCGCGGTAA-3′ and 806RB: 5′-GGACTACNVGGGTWTCTAAT-3′ [46–50]. MiSeq Reagent Kit v2 (2 × 150 cycles, MS-102-2002) was used. Initial sequence processing and analyses were performed using QIIME 2 [51].

### Trial oversight

This clinical trial was supported by the National Institutes of Health. No commercial support was available for this study. All authors participated in data acquisition, had access to data, wrote and/or edited the manuscript, and confirmed the accuracy and completeness of the data.

### Statistical analysis

The recurrence rate of head and neck cancer is 80% over 2 years (50 to 60% local recurrence and 20 to 30% distant recurrence) [4, 5]. In the Phase I trial of PepCan described above the overall histological regression rate of HSILs was 45% [18]. A reduced recurrence rate by the magnitude of 45% would reach 44%. Vaccinating 72 subjects in the PepCan arm and 24 subjects in the placebo arm would have 90% power at two-sided alpha of 5% based on Fisher’s exact test. Accounting for about 5% drop out rate, 75 subjects would be needed in the PepCan arm, and 25 subjects in the placebo arm. The difference between PepCan non-recurrence and PepCan recurrence and between the PepCan and the placebo groups were assessed using a Fisher’s exact test. The 95% confidence intervals were computed using a Clopper-Pearson method. The difference between the injection-related AEs for PepCan and placebo groups were assessed also using the Fisher’s exact test. A two-sided *p*-value of less than 0.05 was used to determine statistical significance, and corrections for multiple comparisons were made where appropriate. A more detailed descriptions of statistical methods are described in the Statistical Analysis Plan.

## SUPPLEMENTARY MATERIALS



## Abbreviations

AE: Adverse event
DLT: Dose-limiting toxicity
TCR: T cell receptor
ELISPOT: Enzyme-linked immunospot assay
HPV: Human papillomavirus
HPV 16: Human papillomavirus type 16
FACS: Fluorescent-activated cell sorting
CTCAE: Common terminology criteria for adverse events
Th1: T-helper type 1
Th2: T-helper type 2
Treg: Regulatory T cell
β-NGF: β-nerve growth factor
CTACK: Cutaneous T cell-attracting chemokine
FGF basic: Eotaxin, basic fibroblast growth factor
G-CSF: Granulocyte-colony stimulating factor
GM-CSF: Granulocyte monocyte-colony stimulating factor
CXCL1: C-X-C motif chemokine ligand 1
HGF: Hepatocyte growth factor
IFN-α2: Interferon-α2
IFN-γ: Interferon-γ
IL-1α: Interleukin-1α
IL-1β: Interleukin-1β
IL-1RA: Interleukin-1 receptor agonist
IL-2: Interleukin-2
IL-2Rα: Interleukin-2Rα
IL-3: Interleukin-3
IL-4: Interleukin-4
IL-5: Interleukin-5
IL-6: Interleukin-6
IL-7: Interleukin-7
IL-8: Interleukin-8
IL-9: Interleukin-9
IL-10: Interleukin-10
IL-12: Interleukin-12
IL-13: Interleukin-13
IL-15: Interleukin-15
IL-16: Interleukin-16
IL-17A: Interleukin-17A
IL-18: Interleukin-18
IP-10: Interferon-γ-induced protein 10
LIF: Leukemia inhibitory factor
M-CSF: Monocyte-colony stimulating factor
MCP-1: Monocyte chemotactic protein 1
MCAF: Monocyte chemotactic activating factor
MCP-3: Monocyte chemotactic protein 3
MIF: Macrophage migration inhibitory factor
CXCL9: C-X-C motif chemokine ligand 9
MIP-1α: Macrophage inflammatory protein1α
MIP-1β: Macrophage inflammatory protein-1β
PDGF-BB: Platelet-derived growth factor subunit B
RANTES: Regulated on activation, normal T-cell expressed and secreted
SCF: Stem cell factor
SCGF-β: Stem cell growth factor β
SDF-1α: Stromal-derived cell factor-1α
TNF-α: Tumor necrosis factor α
TNF-β: Tumor necrosis factor β
TRAIL: Tumor necrosis factor-related apoptosis inducing ligand
VEGF: Vascular endothelial growth factor
APRIL/TNSFS13: Proliferation-inducing ligand
BAFF/TNFSF13B: B-cell activating factor
sCD30/TNFRSF8: Soluble CD30
sCD163: Soluble CD163
gp130/sIL-6Rβ: Glycoprotein 130/soluble interleukin-6 receptorβ
IFN-β: Interferon-β
sIL-6Rα: Soluble interleukin-6 receptorα
IL-11: Interleukin-11
IL-19: Interleukin-19
IL-20: Interleukin-20
IL-22: Interleukin-22
IL-26: Interleukin-26
IL-27, p28: Interleukin-27
IL-28A/IFN-λ2: Interleukin-28A/interferon-λ2
IL-29/IFN-λ1: Interleukin-29/interferon-λ1
IL-32: Interleukin-32
IL-34: Interleukin-34
IL-35: Interleukin-35
LIGHT: Tumor necrosis factor superfamily member 14
MMP-1: Matrix metalloproteinase-1
MMP-2: Matrix metalloproteinase-2
MMP-3: Matrix metalloproteinase-3
OPN: Osteopontin
sTNF-R1: Soluble tumor necrosis factor-receptor 1
sTNF-R2: Soluble tumor necrosis factor-receptor 2
TSLP: Thymic stromal lymphopoietin
TWEAK/TNSFS12: Tumor necrosis factor-like weak inducer of apoptosis
TGF-β1: Transforming growth factor-β1
TGF-β2: Transforming growth factor-β2
TGF-β3: Transforming growth factor-β3
